# Exploring the effects of olfactory VR on visuospatial memory and cognitive processing in older adults

**DOI:** 10.1038/s41598-025-94693-9

**Published:** 2025-03-28

**Authors:** Ryota Sunami, Takamichi Nakamoto, Nathan Cohen, Takefumi Kobayashi, Kohsuke Yamamoto

**Affiliations:** 1 School of Engineering, Institute of Science Tokyo, Yokohama, Japan; 2 Institute of Integrated Research, Institute of Science Tokyo, Yokohama, Japan; 3 Central Saint Martins, University of the Arts London, London, UK; 4 Faculty of Human Studies, Bunkyo Gakuin University, Fujimino, Japan; 5 Faculty of Science and Engineering, Hosei University, Koganei, Japan

**Keywords:** Psychology, Health care, Engineering

## Abstract

This study examined the effects of Olfactory Virtual Reality (VR) Gaming on cognitive performance in older adults. A VR game environment (“Interactive Smellscape”) was created to enable this, and 30 participants aged 63–90 years completed both VR gaming sessions and cognitive assessments, conducted with a 6-day interval between the two sessions. Significant improvements were observed in spatial tasks of Japanese characters and words, with notable enhancements specifically in visuospatial rotation performance and word-location recall accuracy. However, no significant changes were detected in olfactory identification or other general cognitive tasks. These findings suggest potential cognitive benefits of incorporating VR and olfactory stimuli into interventions for older populations, particularly for tasks requiring attention and spatial processing. The results further underscore the importance of task-specific designs to maximize the utility of multisensory VR systems for cognitive rehabilitation.

**Supplementary Information:**

The online version contains supplementary material available at 10.1038/s41598-025-94693-9.

## Introduction

Aging is associated with cognitive declines in memory, attention, and sensory integration, affecting quality of life and independence. As the aging population grows, addressing these declines has become a public health priority. Multimodal interventions combining sensory and cognitive engagement are gaining attention for their potential to enhance brain plasticity and slow cognitive decline. Virtual reality (VR)-based cognitive rehabilitation has shown promise, providing controlled and engaging training experiences^1^.

Among these approaches, olfactory stimulation has drawn interest due to its unique neural pathways, directly linking to memory- and emotion-related regions. Unlike other sensory modalities, which rely on a thalamic relay before reaching higher-order cognitive centers, olfactory signals project directly to limbic structures such as the piriform cortex, amygdala, and hippocampus^2^. This direct connection suggests that olfactory training may support both cognitive and sensory functions through mechanisms distinct from those of visual and auditory modalities^3–5^. Rather than functioning independently, olfactory stimulation complements other sensory inputs, fostering immersive and ecologically valid cognitive engagement. Additionally, olfactory function is an early marker of cognitive decline, making olfactory-based interventions promising for prevention and early detection of neurodegenerative diseases^6^.

Recent studies show that olfactory training can enhance memory performance and induce structural changes in brain regions such as the hippocampus, especially in individuals with mild cognitive impairment^4,5,7–9,]^. Jimbo et al.^10,]^ reported that aromatherapy positively affects individuals with dementia, improving their ability to form abstract ideas and movement and suggested that olfactory stimulation could enhance cognitive function. Similarly, Cha et al.^11^ demonstrated that olfactory training not only improved memory performance but also supported functional recovery in dementia patients, highlighting the potential of structured odor-based therapies in clinical contexts. Birte-Antina et al.^8^ found improvements in olfactory and verbal function in elderly individuals. Furthermore, positive olfactory experiences have been linked to enhanced subjective well-being, which may contribute to both cognitive and emotional resilience in aging populations^12–14^. These findings underscore the potential of olfactory-based cognitive rehabilitation for the elderly.

While traditional cognitive rehabilitation programs primarily rely on visual and auditory stimuli, incorporating olfactory input can enhance sensory integration and engagement by leveraging human cognition’s multimodal nature. Prior research shows that multimodal sensory engagement improves memory retention and learning efficiency more effectively than unimodal sensory experiences^15^. As real-world environments naturally involve multisensory interactions, virtual reality (VR) provides a promising platform to simulate such conditions in a controlled yet engaging manner. By integrating olfactory cues, VR-based cognitive training can better replicate everyday sensory experiences, enhancing ecological validity and facilitating cognitive transfer to real-life situations.

To achieve reliable results, an effective and measurable method for delivering olfactory stimuli must be established. While aroma cards, such as Open Essence (https://labchem-wako.fujifilm.com/jp/category/00368.html), are commonly used for olfactory stimulation, prolonged use can become repetitive and fatiguing. Bauer and Andringa^16^ suggested that immersive VR could be beneficial for cognitive rehabilitation in the elderly due to its flexibility, data collection capabilities, and immersive engagement in cognitive tasks. However, they also emphasized the need for experimental validation with elderly participants.

Moreover, gamification in VR can enhance user motivation and adherence to training programs, addressing a major limitation of traditional olfactory training, which often relies on passive odor exposure in non-engaging environments^17,18^. By incorporating goal-oriented tasks and real-time feedback, VR-based olfactory training can increase cognitive engagement and maximize its therapeutic impact. Studies show that VR-based cognitive training enhances memory, attention, and problem-solving through multisensory engagement^17^. Despite these advancements, few studies have systematically examined the integration of olfactory stimuli into VR-based cognitive rehabilitation, leaving a significant research gap^15^. Thus, further research is needed to determine how olfactory VR can be optimized for cognitive training in aging populations.

While traditional olfactory training programs depend on repetitive and isolated tasks, VR-based interventions provide a more immersive and ecologically valid alternative by simulating real-world scenarios. However, research on the cognitive effects of olfactory VR gaming in older adults remains limited. This study aims to bridge this gap by systematically evaluating its cognitive impact, with a focus on attention, memory, and spatial processing.

We explore the possibility that olfactory VR in an interactive gaming environment could support cognitive rehabilitation. To achieve this, we incorporate multiple cognitive tasks to assess various cognitive functions, identifying which abilities may benefit most from olfactory VR intervention. Additionally, olfactory VR could aid in the early detection of olfactory abnormalities, a known early marker of cognitive impairment. An interactive olfactory game provides olfactory stimuli in a more engaging experience. To this end, we created, developed, and tested an olfactory VR game that simulates natural environments with embedded olfactory stimuli. By integrating olfactory cues into structured cognitive tasks, this approach enhances engagement, motivation, and retention, differentiating it from traditional VR-based cognitive interventions.

For the practical application of olfactory VR, an olfactory display—a device for odor presentation—is necessary. After evaluating various olfactory displays^19–21^, we selected one with multiple solenoid valves, as it effectively dispenses measured quantities of odors within the VR environment and is relatively easy to use^22^.

Earlier examples of olfactory game content include Ranasinghe et al.’s narrative-based game^23^ and Nakamoto et al.’s scent-integrated cooking game^24^. More recently, a collaboration between engineering and art led to the development of an immersive olfactory VR game by Onai and Cohen^25,26^, allowing users to explore and locate smells in natural landscapes. Unlike these studies, which primarily explored immersive experiences, our research is the first to systematically assess the cognitive effects of olfactory VR gaming within a structured cognitive training paradigm for older adults.

This exploratory study aims to investigate the cognitive impact of olfactory VR gaming on memory, attention, and spatial processing in elderly participants. By evaluating this innovative intervention, we aim to contribute to the growing body of research on multisensory cognitive training and its potential applications for aging populations. Specifically, we seek to determine whether structured olfactory VR interventions provide measurable cognitive benefits, thus differentiating our study from prior exploratory VR-based approaches. This is the first report to systematically examine the effectiveness of the "Interactive Smellscape" olfactory VR game for improving cognitive functions in elderly people, expanding on previous VR-based cognitive training research by explicitly integrating olfactory cues into task-driven rehabilitation frameworks.

## Materials and methods

### Devices

Our olfactory VR game consists of four primary components: (a) a high-performance computer running the virtual environment, (b) an olfactory display delivering real-time odor presentation synchronized with gameplay stimuli^24^, (c) a head-mounted display (HMD) (Meta Quest 3, Meta, https://www.meta.com/jp/quest/quest-3/) providing audiovisual feedback and tracking head and body orientation, and (d) VR controllers (Meta Touch Plus Controller, Meta, https://www.meta.com/jp/quest/accessories/quest-touch-plus-controller/) enabling motion-tracked interaction.

The olfactory display, manufactured by Ono Denki (Japan), uses solenoid valves to precisely control odor concentration and release timing. Capable of holding up to 12 distinct odor samples, it enables dynamic odor delivery synchronized with interactive VR elements. Participants engaged with the VR environment while seated in a stabilized swivel chair to ensure safety and accessibility, particularly for those with mobility constraints. To prevent disorientation and accommodate physical limitations, movement was restricted to controller-based interactions, while chair rotation allowed for natural body and head orientation within the VR space.

**Fig. 1:**
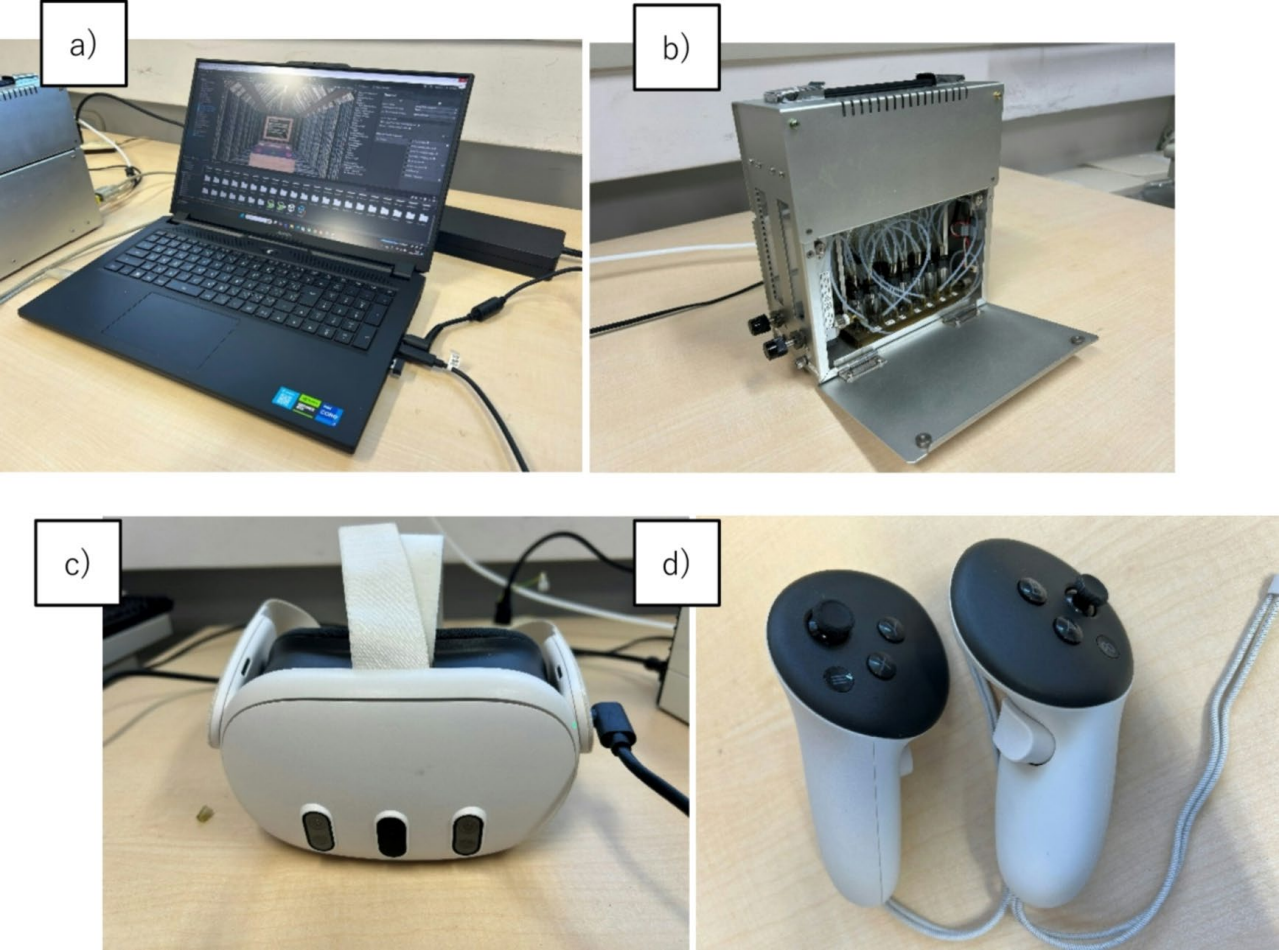
Experimental setup **a**) Laptop computer. **b**) Olfactory Display^22^. **c**) Meta Quest 3. **d**) Meta Touch Plus Controller.

### Interaction with the olfactory game contents

Participants navigated the virtual environment using three primary control mechanisms: walking (left VR controller joystick), touching (both VR controllers), and rotating (natural head and body movement while seated in a swivel chair). These controls were designed for intuitive interaction and to enhance engagement in cognitive tasks. The VR environment features an interactive, navigable landscape where objects emit corresponding olfactory stimuli upon user interaction. Players identify and engage with these objects as they progress through three structured phases, each targeting specific cognitive processes relevant to rehabilitation. The game is calibrated to ensure accessibility and engagement for older adults.

1. Smell and memory initiation phase

**Fig. 2:**
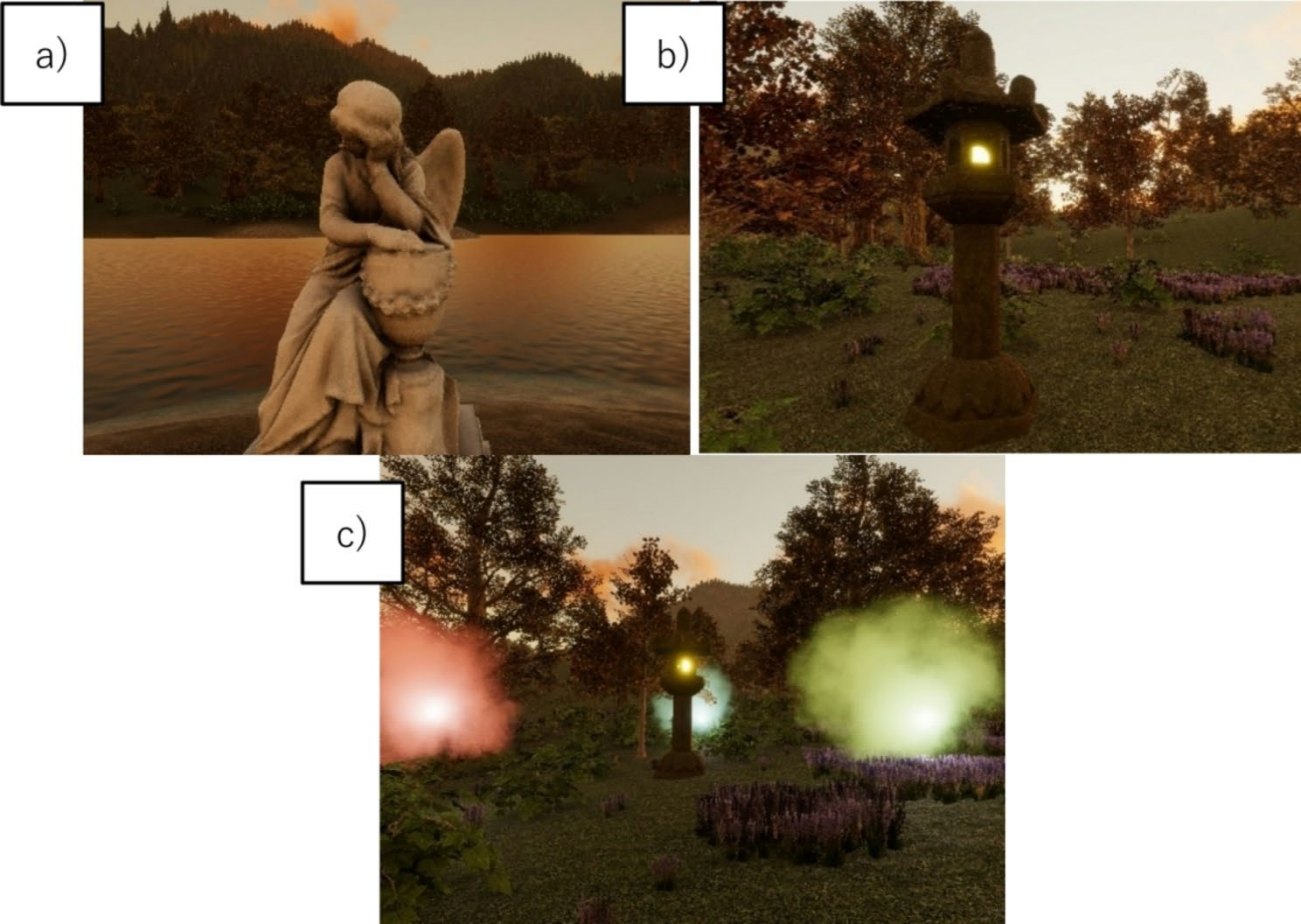
Screenshots of the contents.**a**) smell and memory initiation phase.**b**) Searching for the Odor Source and Maintaining Memory Phase.**c**) Comparison and Selection of the Smell Source Matching Memory Phase.

The game begins when the player touches a virtual statue, triggering the release of a target odor accompanied by a visually paired white cloud lasting five seconds. The goal in this phase is to strengthen odor recognition and memory encoding by linking the olfactory stimulus with a visual cue (Fig. 2(a)).

2. Searching for the odor source and maintaining memory phase

In this phase, players are placed in a predetermined location within a virtual landscape, where they must find a stone garden lantern that acts as the odor source. The odor intensity varies dynamically based on the player’s position and orientation relative to the lantern. Additionally, a flickering light serves as visual guidance.

The primary cognitive challenge is to integrate spatial navigation with odor recognition, while retaining memory of the initially encountered odor stimulus (Fig. 2(b)).

3. Comparison and selection of the odor source matching memory phase

Upon successfully locating the garden lantern, players advance to the odor comparison phase, where they encounter three colored clouds, each emitting a unique olfactory stimulus. The objective is to compare the odors and identify the one matching the initial target odor. Selection is confirmed visually: a correct choice turns the light green, while an incorrect selection turns it red. Players continue distinguishing odor cues until they correctly identify the target odor. This phase engages olfactory discrimination and working-memory retrieval, reinforcing cognitive function (Fig. 2(c)).

### Odors

This study examines odor perception, memorization, and discrimination as core sensory tasks. Prior research indicates that even familiar odors can be difficult to identify without explicit labels^27–29^. Since our VR game excludes verbal labels, odor recognition relies entirely on sensory encoding and memory retrieval, making the task cognitively demanding. To ensure accurate odor discrimination, we selected odors with high perceptual clarity that are easily distinguishable without prior contextual information. These odors were chosen for their ability to elicit strong and consistent olfactory responses across individuals.

Each trial in our game included at least three highly distinguishable odors to ensure valid comparisons. To determine suitable odor sets, we conducted a discrimination triangle test using the olfactory display, with 17 healthy adults (14 males, 3 females) participating. This test identified two significantly distinguishable odor sets:

- Orange, Lavender, Spearmint.
- Melon, Mango, Ume (Japanese plum).

These sets were chosen for their distinct olfactory profiles and minimal perceptual overlap, ensuring clear differentiation across trials.

**Table 1:** Results of the triangle test. A total of 17 participants took part in the test, evaluating a rage of odor pairings. Thirteen participants tested the set comprising orange, lavender,  and spearmint.

Odor 1	Odor 2	Correct	Incorrect	Accuracy (*p*-Value)
(a) Orange, Lavender, Spearmint (13 subjects)				
Orange	Lavender	9	4	0.69 (0.0016)
Lavender	Spearmint	7	6	0.54 (0.035)
Spearmint	Orange	7	6	0.54 (0.035)
(b) Melon, Mango, Ume (11 subjects)				
Melon	Mango	8	3	0.73 (0.0014)
Mango	Ume	7	4	0.64 (0.0088)
Ume	Melon	8	3	0.73 (0.0014)

The results of the triangle tests are presented in Table 1(a) and (b). Statistical analysis confirmed that all odor pairs within each set are significantly distinguishable.

### Participants

Thirty older adults (21 females, 9 males; age 63–90 years, *M* = 71.80, *SD* = 6.14) were recruited from the Iruma Eastern Silver Human Resources Center, a community-based facility for active older adults in Japan. To ensure adequate statistical power, a priori power analysis was conducted using G*Power 3.1. The analysis determined that at least 27 participants were required for a paired two-tailed t-test, assuming an expected effect size of *d* = 0.5 (medium effect per Cohen’s guidelines), a significance level of *α* = 0.05, and a power of *β* = 0.80. The final sample of 30 participants exceeded this threshold, ensuring sufficient power to detect significant effects.

To ensure participant eligibility, a comprehensive health screening was conducted before study enrollment. Since all participants were recruited from a senior employment center, they were generally healthy and functionally independent. This screening assessed general health and nasal function, including olfactory capacity evaluations for anosmia and hyposmia, as these conditions could affect odor perception and confound study outcomes. Eligibility was primarily determined through verbal self-reports rather than clinical assessments. Before enrollment, participants answered screening questions to confirm they had no significant olfactory impairments affecting daily life. Self-reports served as the main criterion for olfactory function screening, ensuring that participants without noticeable impairments were included. Additionally, participants who reported recent respiratory infections, chronic sinusitis, or other conditions affecting olfaction were excluded to prevent transient impairments in odor recognition. The Open Essence olfactory test was administered only to measure potential changes in olfactory function pre- and post-intervention; it was not used for participant selection. Ultimately, 30 participants (aged 63–90) who met these criteria were enrolled. Those who self-reported no significant olfactory difficulties were included, and their data were included in the final analysis.

Prior to participation, all individuals provided written informed consent in accordance with ethical guidelines. The consent form outlined the study’s objectives, procedures, and voluntary nature, ensuring that participants understood they could withdraw at any time without consequences. To enhance comprehension, verbal explanations were provided, and participants were encouraged to ask questions before signing the consent form. This study received approval from the human subject research ethics review committee of Tokyo Institute of Technology (Approval No. A24183) and was conducted in full compliance with the ethical standards of the Declaration of Helsinki.

### Procedure for cognitive task

Participants attended two experimental sessions, scheduled one week apart. In Session 1, they first completed a pre-test battery of cognitive tasks, followed by engagement in the VR odor-based game. During cognitive task evaluation, the experimenter introduced each task with verbal instructions to ensure comprehension. All tasks were paperbased. If necessary, the experimenter offered support for task comprehension while avoiding any assistance that could influence performance. Participants had sufficient time to familiarize themselves with the VR odor game, typically adapting within 5-10 minutes. Some initially reported mild VR-induced dizziness but quickly acclimated, and their interaction with the game became smoother. No participants withdrew or were unable to complete the experiment due to VR sickness. To ensure consistency and control for order effects, all cognitive tasks followed a fixed sequence in both sessions. This methodological approach minimized potential biases from task order randomization. By keeping task order consistent, we reduced performance variability caused by differences in cognitive load or fatigue across participants.

**Fig. 3:**
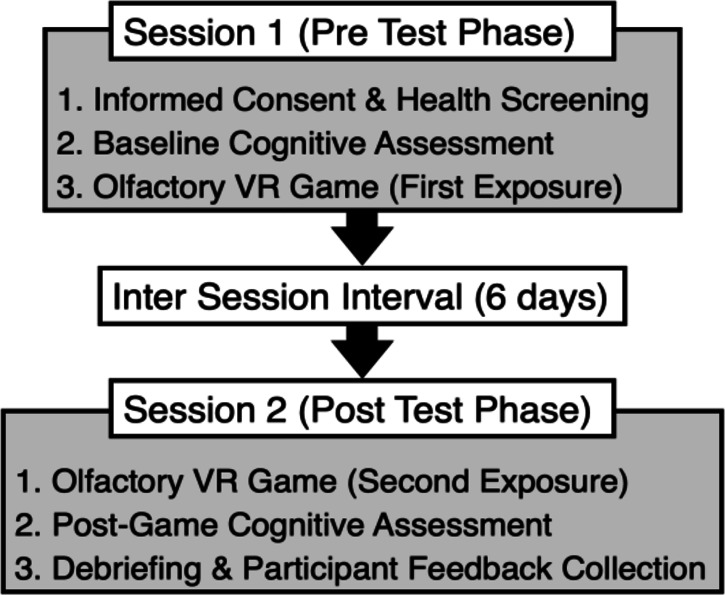
Overview of the experimental procedure.

In Session 2, the sequence was counterbalanced; participants first engaged in the VR game, then completed the post-test cognitive task battery. A five-minute break was included after the olfactory VR game to reduce fatigue before the cognitive tasks. To enhance clarity, Fig. 3 illustrates the experimental design, including participant flow and task sequence. This figure also presents the full session timeline. The specific cognitive task sequence is detailed in the "Cognitive Tasks" section.

This design controlled for order effects and isolated the impact of olfactory VR training on cognitive performance. The VR game followed the same structured scheme described in "Interaction with the Olfactory Game Contents" section and was repeated three times per session to reinforce olfactory learning and memory. While individual completion times varied, the game typically lasted about 20 minutes per session. With cognitive tasks included, total session duration ranged from 60 to 90 minutes, depending on the participant’s pace.

### Cognitive tasks

This study administered multiple cognitive tasks to explore which cognitive functions could benefit from the olfactory VR game. Except for the MMSE and the Hiragana Rotation Task, all tasks were specifically developed for this study, drawing on various cognitive tests to create tailored measures and mitigate ceiling effects. The MMSE was used in its original form, while the Hiragana Rotation Task was adapted from a standardized battery for mild cognitive impairment screening. The remaining tasks were designed by integrating elements from existing cognitive assessments to align with the study’s objectives while ensuring methodological validity. Furthermore, the difficulty levels of the 2D Visual Object Spatial Memory Task and the 2D Word Spatial Memory Task were adjusted to prevent ceiling effects observed in preliminary testing, ensuring sufficient sensitivity to intervention-related changes. To maintain consistent task influence across pre- and post-tests, all tasks followed a fixed sequence, with no randomization between them. This approach prioritized methodological consistency, ensuring that observed changes resulted from the intervention rather than task order variations.

Each task’s structure and its relevance to the study’s objectives are detailed below.

1. Mini-mental state examination (MMSE)

The MMSE was used to assess global cognitive function, evaluating orientation, immediate and delayed recall, attention, and language abilities. Scoring followed standardized protocols, with higher scores indicating better cognitive performance^30^. No time limits were imposed; participants were gently encouraged to continue if they paused while answering.

2. Hiragana rotation task

The Hiragana Rotation Task, adapted from the Perceptual Judgment Continuous Task in the Multiphasic Early Dementia Examination (MEDE), assessed visuospatial processing and perceptual judgment speed. Participants viewed Japanese hiragana characters, each paired with a rotated version (90°, 180°, or 270°). They determined whether the rotated character matched the original, marking “○” for a match and “×” for a mismatch. Since many spatial cognition tasks use shapes or abstract symbols, this task was chosen as a culturally and linguistically appropriate alternative for older Japanese adults. The task lasted one minute, with accuracy (correct responses) and errors (incorrect responses) recorded to assess cognitive flexibility.

3. 2D visual object Spatial memory task

Participants viewed a 3 × 6 grid containing 18 distinct visual objects (e.g., a bird, a microwave, scissors) and had three minutes to memorize their spatial arrangement. After a brief delay, three objects were removed, and participants placed the remaining 15 objects in their original locations on a blank grid. The task had a 10-minute time limit, and performance was scored based on placement accuracy. This task assessed spatial working memory and recall, as spatial memory is particularly vulnerable to aging and dementia. To ensure effective measurement in cognitively healthy older adults, difficulty was increased beyond that of conventional spatial memory tasks. Unlike standard versions prone to ceiling effects, this study used a larger number of objects, requiring participants to encode and retain a more complex spatial configuration. Additionally, the recall phase excluded visual cues, forcing participants to reconstruct the arrangement entirely from memory rather than relying on pattern recognition or partial spatial hints. This enhanced cognitive demand while ensuring feasibility for older adults.

4. 2D word Spatial memory task

This task followed the same procedure as the 2D Visual Object Spatial Memory Task but used item names (e.g., “bird,” “microwave”) instead of images. While the 2D Visual Object Spatial Memory Task assessed spatial recall of concrete visual objects, this task examined spatially associated symbolic memory representation. By requiring participants to encode and retrieve written words within a spatial framework, it evaluated whether the cognitive mechanisms supporting visuospatial memory extend to linguistic-symbolic representations.

Different items were used in each task to prevent interference between tasks. Participants had three minutes to study the grid and a maximum of 10 minutes for the recall phase. To ensure that the task effectively measured verbal spatial memory without inducing ceiling effects, several modifications were introduced. Unlike conventional word recall tasks that depend solely on semantic memory, this task required participants to remember the spatial locations of written words in a 3 × 6 grid. The words were selected to be semantically unrelated, preventing participants from forming associative groupings to aid recall. This approach increased task difficulty by requiring reliance on visuospatial working memory rather than linguistic associations. As in the visual-spatial memory task, no visual cues were provided during recall, necessitating full reconstruction of the spatial layout from memory. This design ensured that the task targeted both symbolic representation and spatial processing, complementing the 2D Visual Object Spatial Memory Task while engaging different cognitive mechanisms.

5. Missing number detection task

Participants were presented with a randomized grid containing numbers from 1 to 30, with two numbers missing. They identified and wrote down the missing numbers. No time limit was imposed, allowing participants to complete the task at their own pace. This task served as a control measure to assess cognitive functions not directly targeted by the olfactory VR intervention. If significant improvements were observed, they could indicate general cognitive benefits rather than VR-specific or olfactory-specific effects. Conversely, minimal changes would suggest that improvements in other tasks were more directly linked to the intervention.

6. Odor identification task

Odor identification ability was assessed using the Open Essence Odor Identification Test (Fujifilm / Wako Chemicals, Japan). Participants smelled a series of standard odors and selected the correct label from multiple choices. The test followed standardized protocols (https://labchem-wako.fujifilm.com/jp/category/docs/00368_pamphlet.pdf). Unlike the other cognitive tasks, this task primarily served to confirm that participants did not have significant olfactory dysfunction, which could confound the main experimental outcomes. Specifically, the test identified extreme cases of olfactory impairment, such as a score of 0 out of 12, or a sudden drop to 0 in Session 2, which might indicate an external factor affecting olfactory function. Additionally, the test ensured that any significant score changes between Session 1 and Session 2 were not caused by external influences unrelated to the intervention.

### Statistical analysis

To evaluate the effects of the olfactory VR game on cognitive performance, statistical comparisons were conducted between pre- and post-intervention scores across all cognitive tasks. Normality was assessed using the Shapiro-Wilk test, and statistical methods were chosen accordingly: paired t-tests were used for normally distributed data, while Wilcoxon signed-rank tests were applied for non-normally distributed data. Effect sizes were computed as Cohen’s *d* for t-tests and *r* for Wilcoxon tests. All data were analyzed without outlier removal to maintain transparency and robustness.

## Results

The statistical comparisons revealed mixed effects across cognitive tasks, with significant improvements observed in some domains, while others remained unchanged. To ensure the robustness of these findings, Shapiro-Wilk tests confirmed that normality assumptions were met for some, but not all, cognitive measures. Consequently, Wilcoxon signed-rank tests were employed for non-normally distributed variables instead of paired t-tests.

**Fig. 4:**
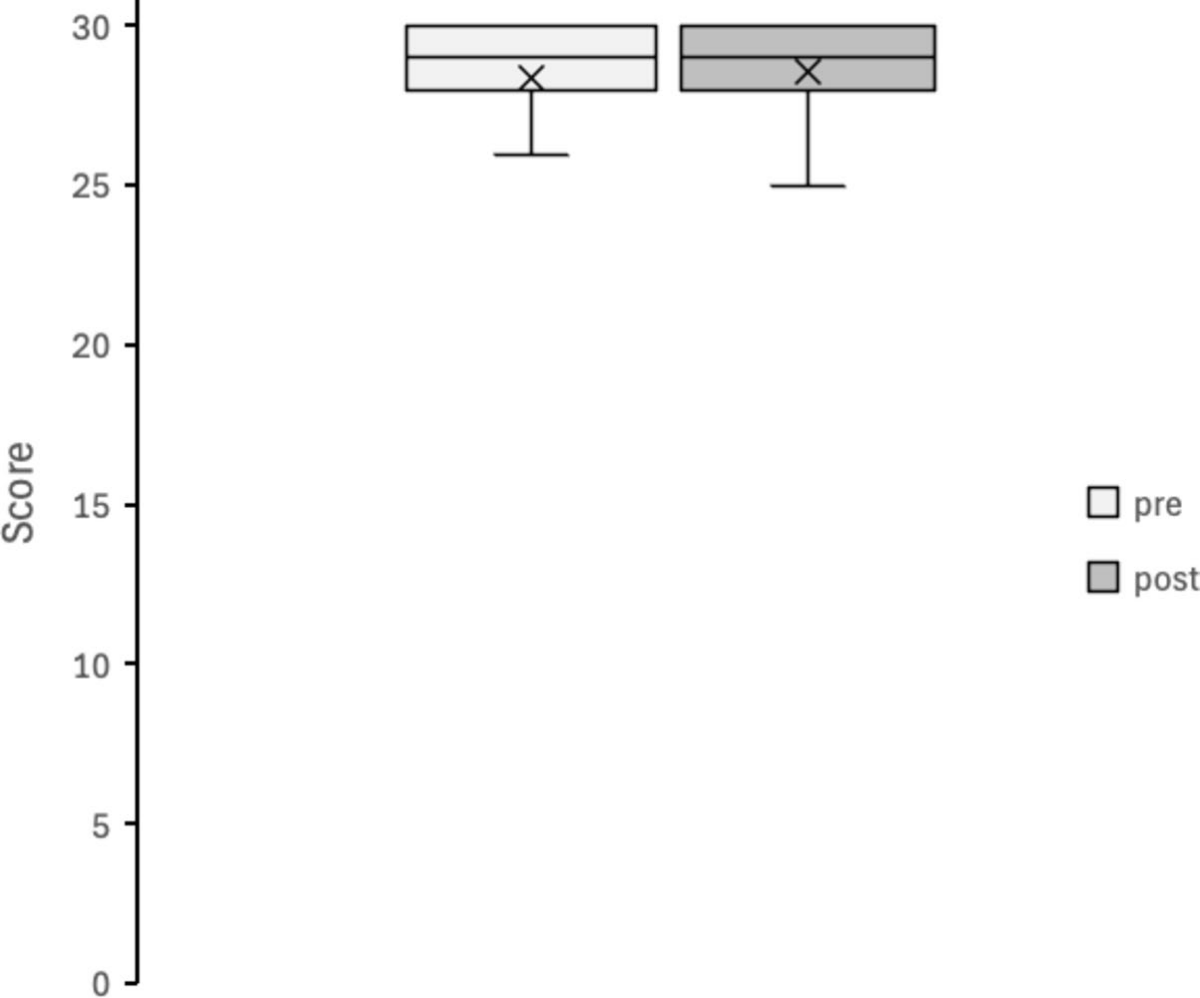
MMSE scores.

**Fig. 5:**
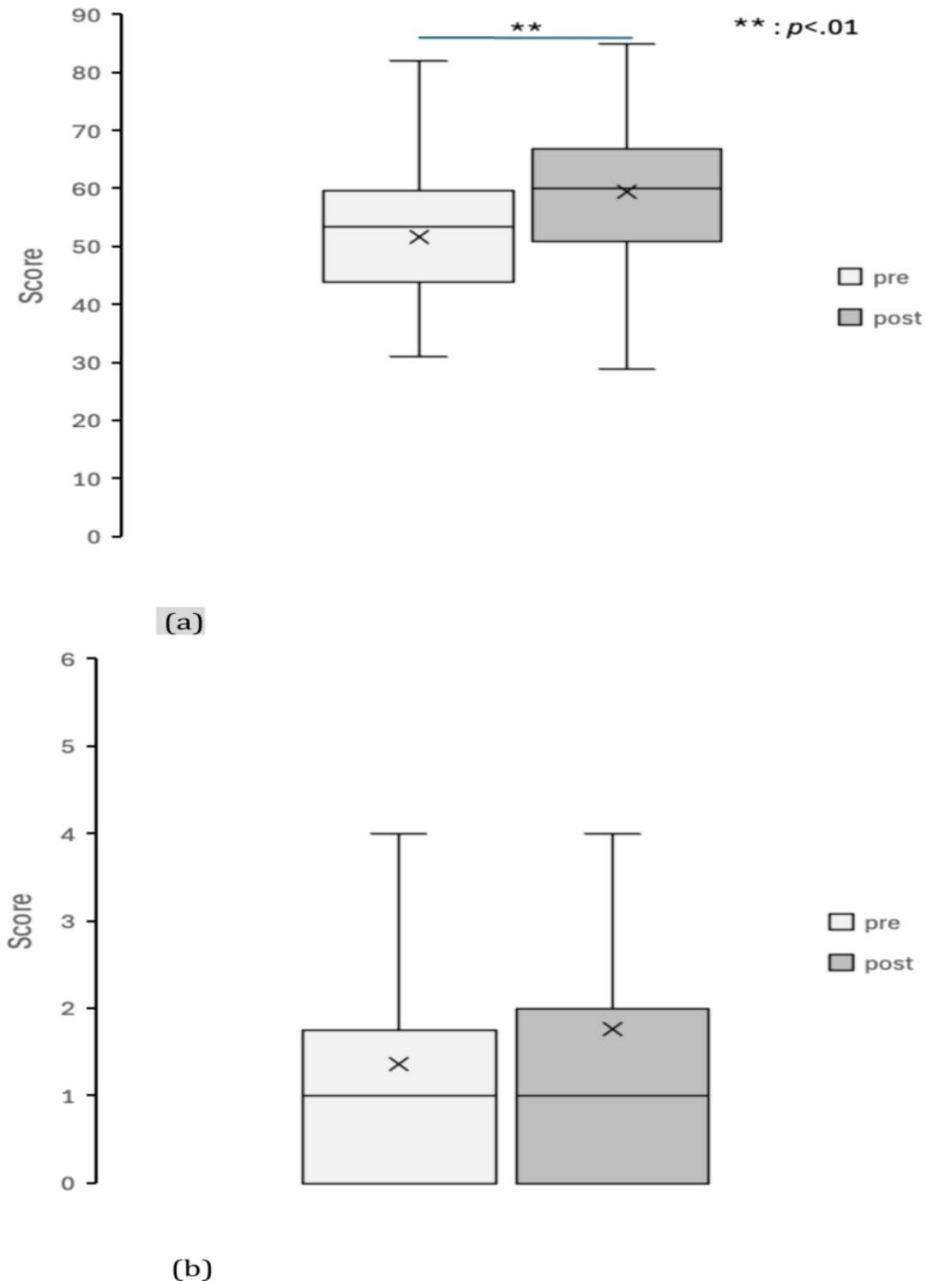
Box plot of scores in Hiragana rotation task (**a**) correct scores and (**b**) error scores.

**Fig. 6:**
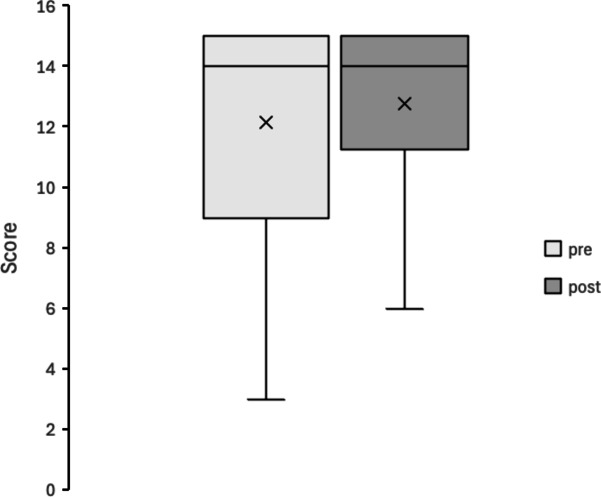
Box Plot of Correct Scores in 2D Visual Object Spatial Memory Task.

**Fig. 7:**
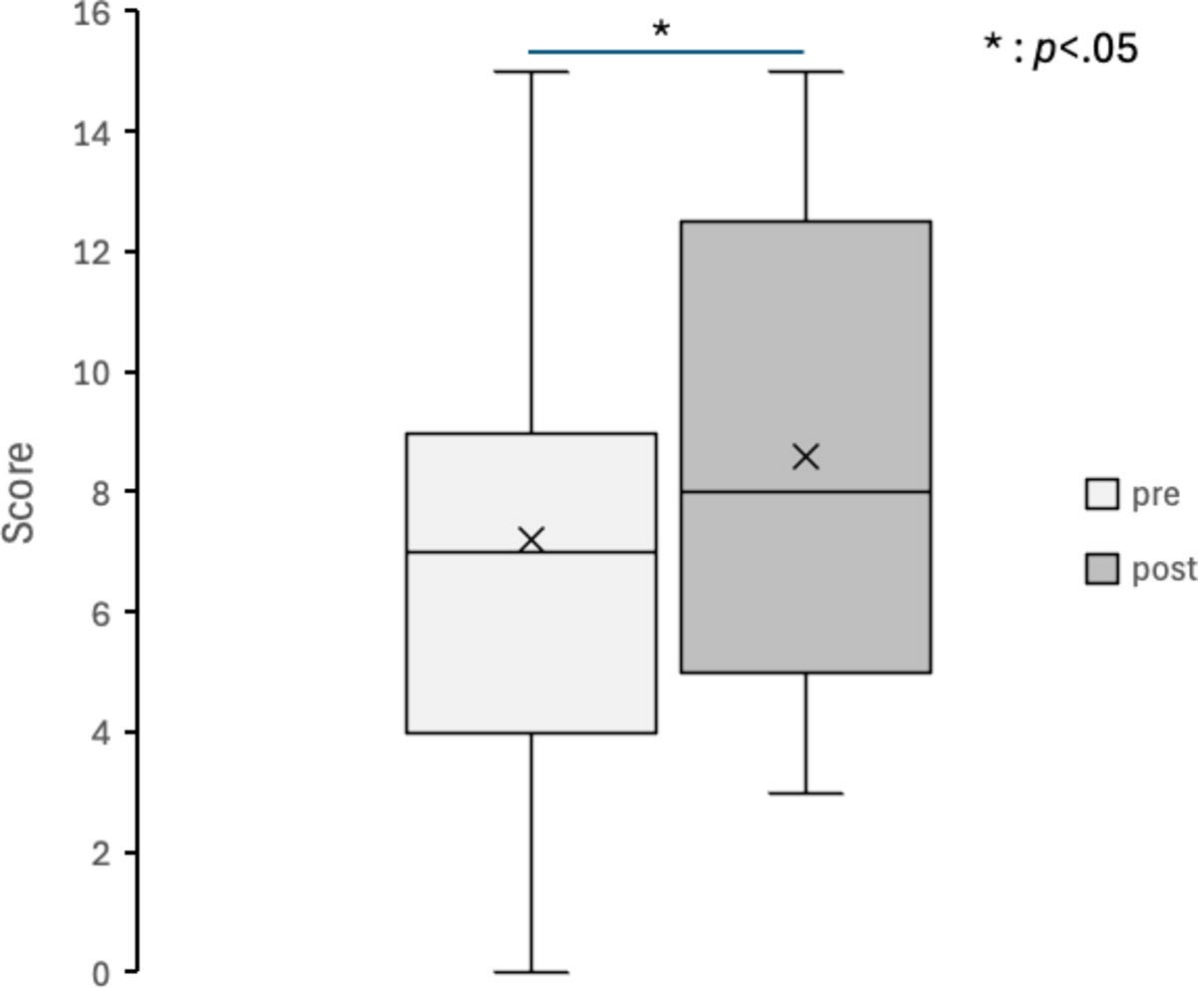
Box Plot of Correct Scores in word spatial memory task.

**Fig. 8:**
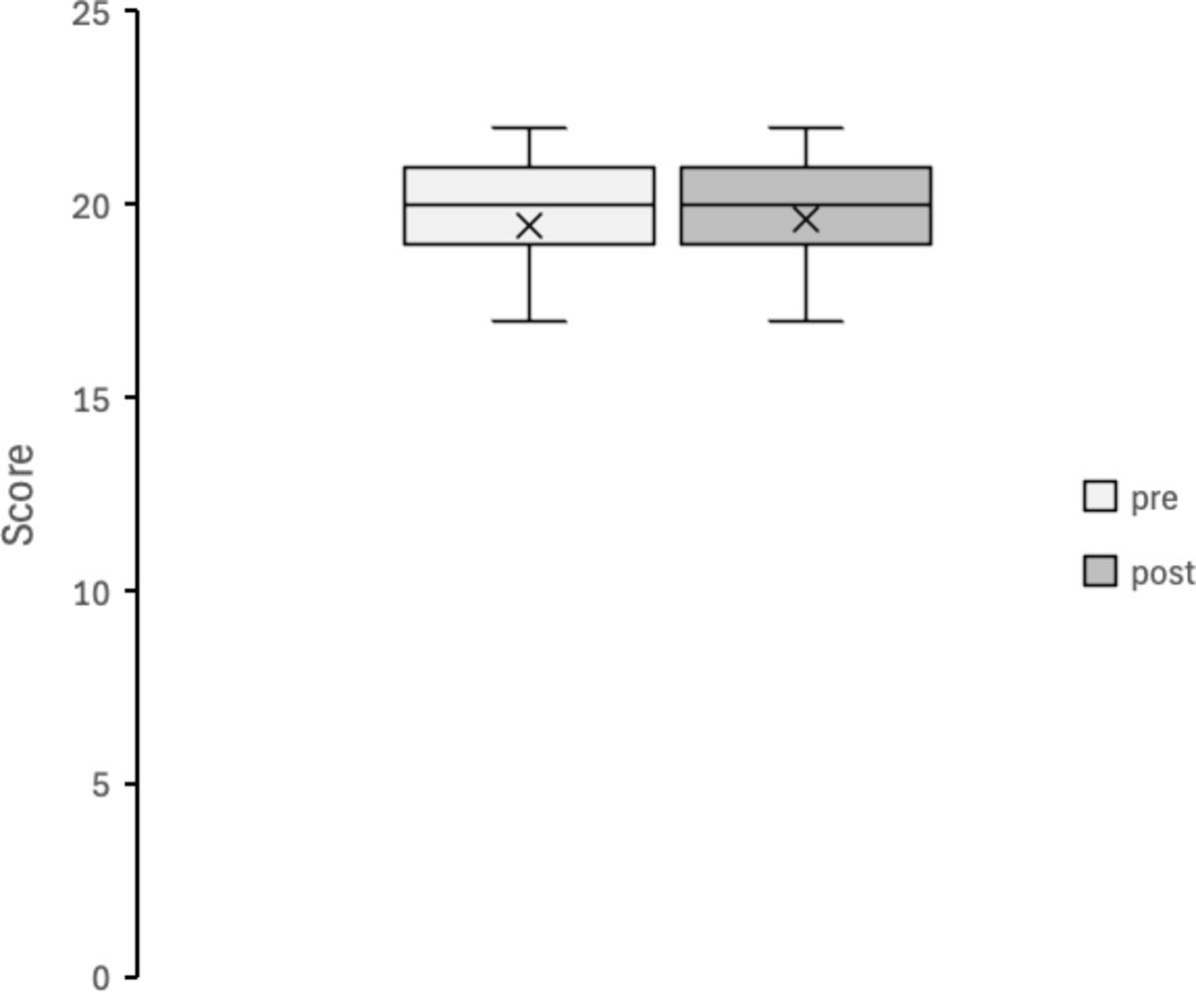
Box plot of correct Scores in missing number detection task.

**Fig. 9:**
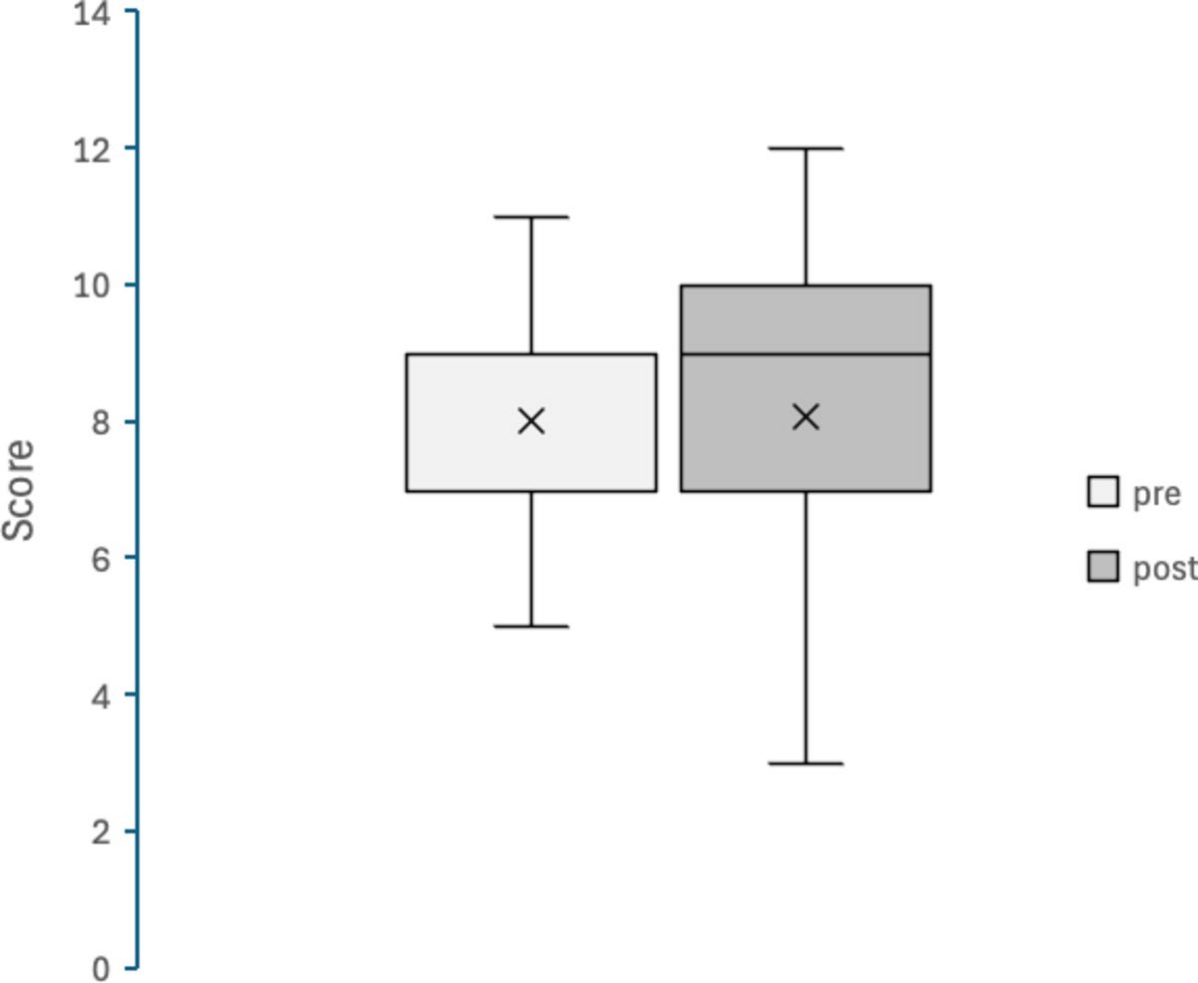
Box Plot of Correct Scores in Odour identification task (Open Essence).

Figures 4, 5, 6, 7, 8 and 9 display box plots illustrating score distributions before and after the intervention. These plots visualize the spread, central tendency, and potential outliers, providing a comparative view of cognitive task performance pre- and post-VR exposure. Additionally, effect sizes and confidence intervals were reported alongside p-values to provide a more comprehensive interpretation of the findings.

The upper edge of the box (Q3, 75th percentile) and the lower edge (Q1, 25th percentile) define the interquartile range. The horizontal line inside the box represents the median (Q2, central value), while the cross symbol (×) denotes the mean. Whiskers extend to the maximum and minimum values within Q3 + 1.5 × IQR and Q1 − 1.5 × IQR, respectively. This plot effectively illustrates the distribution, central tendency, and potential outliers. The “pre” label represents scores before experiencing the Olfactory VR game, while the “post” label represents scores after the intervention.

While the distinction between training effects and the specific impact of the VR intervention requires further investigation, related discussions are presented in the "Discussion" section rather than in the "Results". Additionally, to ensure that the observed changes resulted from the intervention rather than statistical artifacts, all pre-post score distributions were tested for normality. Effect sizes and confidence intervals were reported alongside *p*-values to provide a more comprehensive interpretation of the findings.

1. Mini-mental state examination (MMSE)

Figure 4 shows the distribution of MMSE scores pre- and post-intervention. The box plots indicate that the median and interquartile range remained largely unchanged between conditions. MMSE scores ranged from 23 to 30 pre-intervention and from 25 to 30 post-intervention, suggesting a slight increase but without statistical significance. Statistical analysis confirmed no significant difference in MMSE scores (W = 47.5, *p* = 0.471, *r* = 0.13). This result suggests that the short-term olfactory VR intervention did not produce measurable changes in global cognitive function as assessed by the aMMSE.

pre: session 1 (pre-test phase); post: session 2 (post-test phase).

2. Hiragana Rotation Task.

Figure 5a illustrates the number of correct responses in the Hiragana Rotation Task pre- and post-intervention. The median and upper quartile values increased after the intervention, indicating improved accuracy. Scores ranged from 19 to 82 pre-intervention and from 29 to 85 post-intervention, demonstrating an overall performance increase. The results indicated a significant improvement in the number of correct responses (*t*(29) = −5.88, *p* < 0.001, *d* = 1.07, 95% CI [5.13, 10.60]). Figure 5b shows the number of errors, which remained relatively stable across conditions. Error counts ranged from 0 to 7 pre-intervention and from 0 to 8 post-intervention, indicating minimal variation across sessions. The results showed no statistically significant difference between pre- and post-intervention conditions (W = 51.5, *p* = 0.230, *r* = 0.22). This suggests that the olfactory VR intervention enhanced visuospatial processing and cognitive flexibility in a symbolic rotation task without increasing error rates, which could indicate task fatigue.

pre: session 1 (pre-test phase); post: session 2 (post-test phase).

3. 2D visual object Spatial memory task

Figure 6 presents performance in the 2D Visual Object Spatial Memory Task. The box plots indicate slight variations in individual scores but no substantial shift in the median or interquartile range between pre- and post-intervention conditions. Scores ranged from 3 to 15 pre-intervention and from 5 to 15 post-intervention, suggesting some improvement in lower-performing participants but no major overall change. Statistical analysis confirmed no significant difference (W = 65.5, *p* = 0.381, *r* = 0.16). These results suggest that short-term exposure to olfactory VR did not significantly improve spatial memory for static visual objects. This may indicate that the cognitive effects of olfactory VR are stronger in tasks involving dynamic interaction rather than passive memory encoding.

pre: session 1 (pre-test phase); post: session 2 (post-test phase).

4. Word Spatial Memory Task.

Figure 7 presents the results of the Word Spatial Memory Task. The median and interquartile range of post-intervention scores were higher than pre-intervention scores, suggesting an increase in correct responses. Scores ranged from 0 to 15 pre-intervention and from 3 to 15 post-intervention, indicating an overall performance improvement, particularly among lower-scoring participants. Statistical analysis revealed a significant improvement (*t*(29) = −2.38, *p* = 0.024, *d* = 0.43, 95% CI [0.20, 2.60]). These findings support the idea that multisensory integration, including olfactory cues, can strengthen the encoding and retrieval of symbolic memory representations.

pre: session 1 (pre-test phase); post: session 2 (post-test phase).

5. Missing number detection task

Figure 8 presents the results of the Missing Number Detection Task. The box plots indicate minimal differences between pre- and post-intervention scores, suggesting little change in performance. Scores ranged from 13 to 22 in both sessions, indicating a stable performance level across trials. Statistical analysis confirmed no significant difference (W = 65.5, *p* = 0.380, *r* = 0.16). This task primarily evaluates attention and basic numerical scanning rather than higher-order cognitive processes like memory or executive function. The lack of improvement suggests that olfactory VR did not directly affect attentional processing in numerical tasks, unlike its impact on memory-related tasks.

pre: session 1 (pre-test phase); post: session 2 (post-test phase).

6. Odor identification task

Figure 9 presents the results of the Odor Identification Task. The distribution of scores remained similar between pre- and post-intervention conditions, with no noticeable shift in median values. Scores ranged from 2 to 11 pre-intervention and from 1 to 12 post-intervention, indicating some variability in individual performance but no overall trend toward improvement or decline. Statistical analysis confirmed no significant difference (W = 47.5, *p* = 0.471, *r* = 0.13). This suggests that the intervention did not affect participants’ basic olfactory discrimination ability, reinforcing those cognitive benefits observed in other tasks stem from cognitive mechanisms rather than sensory changes.

pre: session 1 (pre-test phase); post: session 2 (post-test phase).

## Discussion

This study investigated the effects of an olfactory VR game on cognitive functions in older adults, revealing task-specific improvements. Significant gains in the Hiragana Rotation Task and the Word Spatial Memory Task suggest enhanced visuospatial processing and memory recall for characters and words. However, no significant changes were found in in the Mini-Mental State Examination (MMSE), 2D Visual Object Spatial Memory Task, Missing Number Detection Task, or Odor Identification Task, indicating selective rather than broad cognitive benefits.

While this study focused on cognitive functions, prior research suggests that olfactory projections to the limbic system may also influence emotional processes. Given the strong link between memory and emotion, cognitive benefits may have been partly mediated by emotional engagement. Further research should examine how memory recall and attention interact with affective states, incorporating emotional measures such as self-reported mood assessments or physiological indicators.

These findings align with prior research showing that VR-based cognitive training enhances specific abilities, particularly spatial and language-related memory, which require high attention and multisensory integration^1,31,32^. Additionally, odor-evoked autobiographical memory studies suggest that olfactory cues elicit more vivid, emotionally engaging memories than other sensory stimuli, supporting their integration into cognitive training^33^.

The results suggest that olfactory VR interventions are most effective for tasks involving spatial orientation and symbolic processing, rather than for broad cognitive assessments like the MMSE. Future research should examine whether prolonged training or additional sensory modalities enhance these benefits across other domains.

1. Task-Specific improvements and potential explanations

Improvements in the Hiragana Rotation Task and the Word Spatial Memory Task align with prior research showing that VR-based interventions enhance attention, spatial reasoning, and cognitive flexibility^1,31^. Our study’s use of olfactory stimuli may have further strengthened memory encoding by activating the limbic system, particularly the hippocampus and amygdala, which are crucial for memory consolidation^7,34^. Odors, known to evoke autobiographical memories more effectively than other sensory cues^35)^, also influence episodic memory and emotional recall^12,13^. Additionally, olfactory training has been shown to improve working memory, particularly in those with mild cognitive impairment^10,36^, suggesting that olfactory VR may enhance memory-related functions through sensory engagement and neuroplasticity^4^.

A theoretical framework combining multisensory integration, predictive coding, and context-dependent learning explains these findings. The multisensory integration theory posits that the brain optimally integrates sensory information to enhance perception and cognition, particularly when stimuli are congruent and temporally aligned^37,38^. In our study, olfactory cues paired with spatial tasks may have improved attentional focus and encoding efficiency, enhancing memory recall. Additionally, VR-based cognitive training research suggests that improvements in visuospatial tasks and cognitive flexibility may stem from VR’s immersive properties. VR has been shown to enhance visuospatial processing and decision-making, especially in tasks requiring real-time spatial adjustments^39^. This aligns with our finding that the Hiragana Rotation Task and the Word Spatial Memory Task, which require spatial transformations and working memory coordination, showed significant gains. Moreover, VR interventions tend to benefit cognitively demanding, interactive tasks, explaining why improvements were observed in specific tasks but not in global cognitive measures like the MMSE^40,41^.

Predictive coding theory suggests that the brain continuously generates expectations about sensory input and updates them based on discrepancies between expected and actual stimuli^42^. In odor-spatial matching tasks, participants likely anticipated scent-location associations, refining predictions through feedback. This may have facilitated more efficient spatial encoding, contributing to observed memory improvements. Interestingly, while some visuospatial functions improved, MMSE scores and Object Spatial Arrangement tasks showed no significant gains. This aligns with findings that VR-based interventions are more effective for cognitively demanding, dynamic tasks than for standardized cognitive measures^40,41^. VR enhances task-specific abilities, especially those involving real-time interaction and problem-solving, but its effects on broader cognitive scales remain inconsistent^41^, reinforcing the role of task nature in VR training effectiveness.

Context-dependent learning suggests that memory retrieval improves when encoding-context cues reappear during recall^43^. Since olfactory stimuli link directly to the hippocampus and limbic system, they may have served as strong contextual anchors, enhancing recall by reinforcing spatial associations.

Together, these models provide a coherent explanation for the observed cognitive benefits, suggesting that olfactory VR training enhances learning through predictive mechanisms, cross-modal integration, and contextual reinforcement. Future research should refine its application by manipulating sensory congruency, prediction errors, and retrieval contexts. Additionally, examining cultural influences on VR training effectiveness is crucial for broader applicability.

However, our findings emphasize the task-dependent nature of VR-based cognitive enhancements. While tasks involving active spatial manipulation and cognitive flexibility improved, those assessing general cognition or static spatial arrangement did not. This distinction is crucial for future research, indicating that VR cognitive training should target specific functions rather than assume uniform benefits^39,40^. Additionally, Willander et al.^44^ found that olfactory cues alone evoke more vivid, emotionally engaging autobiographical memories than when paired with visual stimuli. This suggests that multimodal integration is not always beneficial and should be considered in relation to task demands and sensory interactions.

Although our study did not directly assess olfactory dysfunction, we accounted for variability in participants’ olfactory function. All were generally healthy, reporting no subjective impairments affecting daily life. Given the broad age range (63–90 years), some variation in odor identification was expected. Self-reported olfactory function served as the primary inclusion criterion, ensuring participants had no noticeable deficits. While some showed relatively low Open Essence scores, these reflected weaker odor intensity rather than complete loss. Importantly, all actively engaged in odor-based exploration during the VR game, confirming their ability to process olfactory cues. Thus, all data were valid and included in the final analysis.

These findings suggest that task-specific improvements may result from the synergy between VR-based cognitive engagement and olfactory processing. However, while olfactory VR shows potential cognitive benefits, these results remain preliminary. Given the short-term nature of our study, it is unclear whether improvements reflect temporary learning or lasting neuroplastic changes. Although VR interventions engage multiple sensory and cognitive processes, our study does not establish their direct impact on brain structure. Further research with neuroimaging or longitudinal designs is needed to assess the long-term effects of olfactory VR on cognition.

Given the strong link between olfactory processing, memory, and emotion, future studies should examine whether cognitive improvements also enhance emotional well-being, such as reducing stress or increasing positive affect. Investigating the cognitive-emotional interaction in olfactory VR could provide a deeper understanding of its benefits for aging populations. Additionally, assessing whether prolonged olfactory VR training sustains cognitive improvements and mitigates age-related decline would be valuable.

2. Challenges in disentangling training effects and intervention impacts

A key challenge in this study was distinguishing VR-based cognitive gains from general training effects. While practice effects may have contributed, the lack of significant changes in the MMSE, Odor Identification Task, and 2D Visual Object Spatial Memory Task suggests that improvements in the Hiragana Rotation Task and the Word Spatial Memory Task were not solely due to repetition. This aligns with prior findings that VR selectively enhances specific cognitive domains rather than uniformly improving all tasks^1,31,32^.

Olfactory VR interventions may offer a novel pathway for enhancing cognitive function beyond traditional training by promoting neural plasticity. Olfactory training has been shown to induce structural changes in the hippocampus, a key region for memory^5^. These neuroplastic effects suggest that integrating olfactory stimuli into VR training could benefit older adults. However, given that this study was a short-term intervention, it remains unclear whether significant plasticity changes occurred. Further research with extended training and neuroimaging is needed to assess these effects.

To better isolate VR-specific benefits from practice effects, future studies should implement randomized controlled trials (RCTs) with longitudinal follow-ups and control conditions comparing VR with and without olfactory stimuli. This approach would provide stronger evidence for the unique contributions of olfactory cues to cognitive improvement.

3. The role of olfactory stimulation in cognitive engagement and emotional processing

The Hiragana Rotation Task and other mental rotation tasks engage visuospatial reasoning and memory^31,32^, requiring participants to generate and manipulate spatial representations. These processes involve both controlled and automatic cognitive resources^45^. Initially, controlled processing dominates unfamiliar tasks, but with practice, participants shift to more efficient, automatic processing^46^. Improvements in the Hiragana Rotation Task suggest that VR-based cognitive engagement, combined with olfactory cues, may facilitate this transition from effortful control to fluent processing.

Olfactory cues in VR interventions enhance immersion, memory recall, and emotional responses^12,34,47^. The “Proustian memory” concept, reviewed by Hackländer et al.^33^, highlights olfactory stimuli’s unique role in triggering emotionally rich, long-term memories, potentially explaining memory-related cognitive enhancements. The Proust effect, described by Yamamoto & Sugiyama^13^, further supports this, showing that olfactory stimuli evoke autobiographical memories more effectively than other sensory modalities.

Olfactory stimuli enhance behavioral realism in virtual environments. Khan & Nilsson^48^ found that scent cues, such as fire smells, significantly enhanced user responses in VR simulations. This suggests that olfactory VR could create more ecologically valid training environments, improving immersion and effectiveness.

These effects were likely amplified in the current VR environment through multisensory stimulation and neuroplastic adaptation. The well-documented connections between the olfactory system and brain regions like the hippocampus and amygdala^2,7^ may explain how olfactory stimuli enhance memory consolidation and spatial processing. Additionally, the emotional salience of certain odors may reinforce cognitive engagement and increase task motivation in older adults.

Given these findings, future research should explore how different olfactory stimuli selectively influence cognitive and emotional processing in VR training. Studies should examine whether odor categories (e.g., familiar vs. novel, neutral vs. emotionally charged) differentially affect attention, memory, and decision-making. This could enable targeted interventions for cognitive decline, leveraging olfactory stimulation to optimize performance and enhance emotional well-being.

4. Cultural considerations, task adaptation, and future directions for implementation

The Hiragana Rotation Task was designed for Japanese participants, using hiragana to assess mental rotation and spatial reasoning. However, its reliance on the Japanese writing system limits its applicability to non-Japanese populations. In other cultural contexts, mental rotation tasks using rotated alphabets, geometric shapes, or culturally neutral symbols effectively assess similar cognitive domains. For example, Kail & Park^49^ demonstrated practice effects in letter orientation judgment tasks, validating their use for evaluating mental rotation and processing speed. Similarly, Suzuki et al.^50^ used mental rotation-based tasks to screen for mild cognitive impairment, highlighting their potential for assessing visuospatial functions across cultures. As these studies were conducted in different cultural settings, future research should explore how writing systems and character symbolism (e.g., logographic vs. alphabetic scripts) influence cognitive processing and task performance.

Given the challenges of deploying olfactory VR in real-world settings, our interdisciplinary collaborations with experts in engineering, art, psychology, industry, and healthcare aim to develop scalable, cost-effective solutions for cognitive rehabilitation. Recent advancements show the feasibility of integrating olfactory displays into VR for applications from rehabilitation to immersive training^19,22^. However, technological and economic barriers persist, particularly in scalability, portability, and affordability for clinical and commercial use.

VR-based cognitive training must be tailored to different user groups, considering cultural variations, cognitive abilities, and sensory preferences. Andonova et al.^47^ found that olfactory VR effectiveness varies with prior sensory experiences and learning contexts. This underscores the need for culturally and cognitively adaptive VR training protocols that accommodate diverse sensory interactions.

Future studies should incorporate culturally adaptable cognitive tasks, such as alphabet-based mental rotation, non-language spatial reasoning, and real-world navigation, to improve cross-cultural applicability^51^. Additionally, usability studies across diverse age groups, cultures, and sensory abilities are needed to refine cognitive training and optimize engagement.

5. Limitations and future directions

This study has several limitations. First, the lack of a control group without olfactory input prevents definitive causal attribution of cognitive improvements to the olfactory VR intervention, leaving uncertainty about whether effects stemmed from VR exposure, olfactory stimulation, or practice effects. Future studies should implement randomized controlled trials (RCTs) to strengthen causal inferences^1^. Although task-specific improvements were observed, the MMSE and the 2D Visual Object Spatial Memory Task showed no significant changes, suggesting benefits were limited to specific tasks rather than broad cognitive enhancements^1,31,32^. Future research should use larger samples and randomized controlled designs to further validate these effects and explore underlying mechanisms.

Second, the short intervention may have limited its ability to produce sustained cognitive improvements. Prior research suggests that prolonged training is needed for lasting neural adaptations, especially in those with mild cognitive impairment^4,5^. Longitudinal studies should evaluate whether cognitive benefits persist beyond a single intervention and explore olfactory VR’s potential for long-term cognitive maintenance.

Third, individual differences in olfactory sensitivity may have influenced cognitive outcomes. Although all participants self-reported intact olfactory function, age-related declines and variations in perception could have affected cognitive responses to odors^52^. Future interventions should personalize odor intensity based on individual thresholds to optimize olfactory VR training effectiveness.

Fourth, while the MMSE is a widely used cognitive screening tool, it lacks sensitivity to detect subtle intervention-related changes, especially in task-specific improvements. Future studies should use more sensitive tests, such as the Montreal Cognitive Assessment (MoCA) for executive function or the Rey-Osterrieth Complex Figure Test for visuospatial processing. Additionally, the Missing Number Detection Task lacked time constraints, possibly reducing its sensitivity to subtle improvements. Introducing timed conditions or adaptive difficulty could enhance measurement precision and prevent ceiling effects.

These findings suggest that olfactory VR selectively enhances specific cognitive domains, particularly spatial memory and attention, while having limited effects on global cognition. This aligns with research on multisensory integration, which shows that sensory-based interventions impact cognitive domains differently^53^. Further exploration of sensory interactions in VR is needed to optimize intervention design.

Practical implementation remains challenging due to the cost, accessibility, and complexity of olfactory display systems. Collaboration with engineers and industry partners is crucial to developing scalable, cost-effective solutions for clinical and elderly care^16^. Future research should explore alternative odor delivery methods, such as compact diffusion systems, to improve feasibility for large-scale use.

Cultural differences in sensory processing and memory recall underscore the need for culturally adapted VR interventions. Research suggests that olfactory memory recall varies across populations^14^, indicating that cultural factors may influence intervention efficacy. Future studies should include diverse participant groups and examine how linguistic and cultural differences impact engagement with VR-based cognitive training.

## Conclusion

This study demonstrates that incorporating olfactory stimuli into VR-based cognitive training can enhance specific cognitive functions, particularly visuospatial processing and memory recall. The observed improvements in the Hiragana Rotation Task and Word Spatial Memory Task suggest that multisensory stimulation, particularly olfactory input, facilitates cognitive engagement and task performance in older adults. Although some improvements may be influenced by practice effects, the findings underscore the critical role of task design—including time constraints, task type, and complexity—in shaping cognitive outcomes. These results align with prior research on multisensory integration and olfactory training, reinforcing the potential of olfactory VR as a novel tool for cognitive rehabilitation.

While further research is needed to fully establish its efficacy and long-term benefits, these findings add to the growing body of evidence supporting multisensory VR interventions for aging populations. Future studies should focus on optimizing task design, evaluating long-term cognitive effects, and developing scalable olfactory VR solutions for real-world applications.

## Electronic supplementary material

Below is the link to the electronic supplementary material.

Supplementary Material 1

## Data Availability

All data analysed during this study are included in this published article and its supplementary information files.
